# Modified Extended Kalman Filter and Long Short-Term Memory-Based Framework for Reliable Stride-Length Estimation Using Inertial Sensors

**DOI:** 10.3390/s26041096

**Published:** 2026-02-08

**Authors:** Qian Mao, Fan Yang

**Affiliations:** 1 School of Design, The Hong Kong Polytechnic University, Hong Kong, China; 2 Department of Applied Physics, The Hong Kong Polytechnic University, Hong Kong, China

**Keywords:** gait and balance, global aging, extended Kalman filtering, modified extended Kalman filtering

## Abstract

Gait analysis plays a critical role in assessing mobility and identifying risks such as frailty and falls, where accurate spatiotemporal measurements are essential for early intervention, particularly in aging populations and clinical screening contexts. However, robust gait characterization remains challenging due to noise contamination and variability in sensor-based signals. To address these limitations, this study presents a stride-length estimation framework formulated as a modified processing-and-estimation pipeline integrated with Long Short-Term Memory (LSTM) networks. The pipeline includes wavelet-based denoising and cubic-spline interpolation as front-end preprocessing, followed by a Kalman-filtering stage with dynamic gain regulation guided by acceleration zero-crossing events to mitigate transient errors around abrupt turning points. Experimental data were collected from twelve healthy participants (seven females, mean age: 26.76 ± 3.01 years; five males, mean age: 25.81 ± 1.63 years) walking at self-selected speeds on a treadmill, using both an inertial sensor-based gait monitoring system and a motion capture system as the ground-truth reference. The proposed framework demonstrated a substantial improvement in stride-length estimation accuracy, reducing the absolute mean error from 29.78% to 7.77% and the standard deviation from 20.31 to 7.17. Furthermore, the LSTM models trained on Modified EKF-preprocessed data achieved superior performance metrics, with a Mean Absolute Error (MAE) of 0.0376 and a coefficient of determination (R^2^) of 0.7066. These results highlight the effectiveness of combining Modified EKF preprocessing with LSTM learning to enhance stride-length estimation reliability. This integrated approach offers a robust, noise-resilient solution for wearable gait analysis, providing valuable insights for clinical diagnostics, rehabilitation monitoring, and health management applications.

## 1. Introduction

Population aging is accelerating worldwide, increasing the demand for objective and scalable assessments of mobility and functional health [1]. Timely monitoring of gait-related indicators is particularly relevant for fall-risk screening and rehabilitation-oriented care [2,3]. Wearable inertial sensors provide a practical means to capture locomotion signals in daily-life settings; however, reliable estimation of spatiotemporal parameters such as stride length remains challenging due to measurement noise, signal variability, and non-stationary walking dynamics [4]. These challenges motivate the development of robust signal-processing and estimation pipelines for stride-length analysis from inertial measurements.

Falls are the second leading cause of premature death in the elderly, which could also adversely affect their physical and normal life [5]. Thus, devices for assistive walking, sensing gait, and even physical training therapy have been explored and adopted as the main means of preventing falls in the elderly. Among the aforementioned methods, gait monitoring with sensors during walking is a low-cost and mobile solution that is crucial for preventing falls in the elderly and has promising growth prospects [5]. Cardiovascular and neurodegenerative diseases, such as heart disease, stroke, and dementia, are well-established risk factors for falls in older adults [6,7,8]. Researchers have increasingly focused on wearable sensing technologies as tools for gait assessment and fall-risk monitoring in older adults [6]. By continuously capturing locomotion signals, wearable sensors enable the quantification of gait characteristics and the detection of changes in stability over time, which can support clinical decision-making and rehabilitation or preventive interventions [9]. Nevertheless, measurement noise and motion artifacts can degrade the accuracy of sensor-derived signals. Filtering approaches, such as Kalman-based methods, can mitigate noise effects and improve the reliability of the estimated gait parameters [10]. Although the target application is motivated by aging-related mobility risks, the proposed method is validated on inertial gait data collected from healthy adults in a controlled evaluation setting, providing a basis for subsequent deployment in clinical and at-risk populations.

Gait analysis can be used to assess the walking function of the human body objectively and quantitatively in the processes of diagnosis, rehabilitation assessment, and efficacy evaluation of related diseases [11]. Gait information provides rich quantitative digital descriptors of human movement, which are typically summarized by spatiotemporal, kinematic, and kinetic parameters [12,13]. In practical wearable sensing scenarios, the estimation of these parameters is often affected by measurement noise, signal variability, and cumulative errors, which can degrade stability and accuracy over time. Consequently, parameter adjustment or adaptive correction mechanisms are frequently introduced to mitigate error accumulation during signal processing or model inference. For example, adaptive feedback strategies based on multi-feature collaboration have been employed in noise-robust LSTM frameworks to adjust model parameters and improve estimation consistency under noisy conditions [14]. In addition, real-time correction is proposed and implemented for time-varying characterization and calculation. Coordinated transport optimization is then applied using a feature spiral-up strategy, demonstrating the feasibility of multi-parameter correction for real-time estimation [15]. Stride length, as a spatiotemporal marker, is associated with fall risk and can support gait assessments and risk monitoring [16]. The stride length refers to the longitudinal distance between the heel (or toe) of the foot [11]. Changes in stride length were associated with the risk of falls and cognitive impairment in older adults [13]. Accurate stride-length information can support quantitative gait assessments and functional outcome monitoring in clinical and community settings [17]. Therefore, it is essential to investigate how to accurately obtain the complementary stride length.

The estimation of a pedestrian’s stride length presents a formidable challenge, necessitating the employment of diverse methodologies contingent on the precise placement of sensors. Various step-length estimation algorithms were assessed for pedestrian inertial estimation. The findings indicated that the sensor’s mounting location had a negligible impact on the performance of these estimators [18]. The angular velocity of the thigh in a single leg was measured using a piezoelectric gyroscope, and the results showed that in a sample of twenty healthy persons, the relative accuracy was about ±15% [19]. Furthermore, the development of algorithms for extracting precise data from sensors and accurately estimating stride lengths is of paramount importance in the realm of computer science.

The rapid development of accelerometers and gyroscopes in recent years has provided novel approaches for the acquisition of gait data [20]. The use of portable terminals with acceleration sensors combined with algorithms to measure and predict the stride length is the main approach at present, while the algorithms include least-mean-squares methods, Kalman-filtering algorithms, machine learning approaches, etc. [21]. Machine learning requires a large amount of data to train the model, which makes real-time estimation difficult with a high error rate [22]. The advantage of the Kalman filter algorithm over the least-squares method is the involvement of noise influence during sensor measurements in the calculation process, thus enabling the calculation results to be closer to the real data [23]. Although Kalman filtering provides an effective means for estimating stride length, significant errors can still arise during walking due to signal distortions caused by noise interference [24]. Such inaccuracies highlight the limitations of conventional Kalman filtering in handling real-world inertial sensor data.

To address this gap, the proposed framework explicitly establishes a link between physics-based sensing and deep learning by using a Modified Extended Kalman Filter (MEKF) to transform raw inertial measurements into physically meaningful, uncertainty-aware state estimates, which are then organized as structured temporal sequences for LSTM. In this way, the MEKF serves as a model-based “physical perception” front end that constrains and regularizes the subsequent learning stage, while the LSTM captures residual nonlinearities and temporal dependencies that are difficult to represent with fixed parametric motion models. Notably, many emerging Kalman/EKF-based gait estimators still operate with predefined noise assumptions and limited adaptability, and they do not fully exploit the flexibility of deep learning-embedded representations. By contrast, our MEKF–LSTM design emphasizes this embedding interface, enabling a more modular integration between state-space estimation and data-driven sequence modeling.

The contributions of this work are as follows: First, we present a physics-informed learning pipeline that couples a Modified Extended Kalman Filter (MEKF) with LSTM by using the MEKF to convert raw footbed inertial measurements into physically interpretable, uncertainty-aware latent states, which are then used as the sequential input to the LSTM for stride-length estimation. This design provides a clear and reproducible interface between state-space estimation and data-driven temporal modeling. Second, we specify the MEKF formulation in full, including the state definition, the state-transition model and its associated matrix representation, and the measurement model that links inertial signals to the estimated states, thereby improving methodological transparency and reproducibility. Third, we implement and evaluate an end-to-end system—signal denoising, gait-event/stride segmentation, recursive MEKF estimation, and LSTM-based sequence learning—and show that the proposed approach yields lower stride-length estimation error than a conventional Kalman filter baseline under the same sensing configuration and experimental protocol.

The inertial sensor embedded in the footbed is used to record foot acceleration signals during walking. To mitigate measurement noise and improve signal continuity, we apply wavelet-based denoising and cubic-spline interpolation prior to estimation. The preprocessed inertial signals are then fed into the proposed MEKF–LSTM framework to estimate stride length. The improved estimation accuracy relative to the conventional Kalman filter baseline suggests that the proposed framework can support wearable gait monitoring applications relevant to mobility assessments and fall-risk screening.

## 2. Methodology

### 2.1. Data Collection

Twelve healthy young adults were enrolled in the study, seven of whom were female (26.76 ± 3.01 years old) and five of whom were male (25.81 ± 1.63 years old). On a treadmill, participants were instructed to walk at their own pace. Data were collected concurrently using both the gait monitoring system and the motion capture system. The gait monitoring system employed a footbed-mounted inertial sensor (WT901BLE5.0C) to gather tri-axial acceleration and angular velocity signals (and the sensor provided attitude/angle estimates), which were subsequently recorded and analyzed using the modified Kalman-filtering algorithm. To assess the effectiveness and consistency of the gait monitoring system, motion data of participants were also captured using the Vicon system (Oxford Metrics, Oxford, UK). This allowed for the comparison of stride-length variations obtained from the gait monitoring system against those derived from the Vicon system, serving as a benchmark for usability and reliability assessments.

### 2.2. Modified Extended Kalman Filter and Long Short-Term Memory-Based Framework

As sensors, even the accurate ones provide data with the presence of noise [25]; an algorithm for optimization to exclude the noise interference of sensors, such as the Kalman filter algorithm, is needed to estimate the stride length. The Kalman filter algorithm is a recursive algorithm that uses the state-space method to design the filter, making it suitable for the estimation of multidimensional stochastic processes [23]. Kalman filtering realizes the optimal estimation of the real value based on the consideration of noise interference [26]. The process noise comes from the uncertainty of the system dynamics model, and the measurement noise mainly comes from various disturbances generated in the process of measurement [27].

In this study, a processing pipeline combining wavelet-based denoising, cubic-spline interpolation, and Kalman filtering is implemented for stride-length estimation from inertial signals. Wavelet denoising is first applied to suppress high-frequency noise components in the measured acceleration signals, followed by spline interpolation to improve signal continuity. The wavelet transform is formulated using a finite-length decaying wavelet basis, as defined in Equations (1)–(6). Through multiscale decomposition, signal and noise exhibit distinct characteristics across different scales, enabling effective noise attenuation prior to state estimation. Based on the preprocessed signals, the Kalman-filtering stage is then formulated using the state-space representation given in Equations (7)–(13), which describe the state transition and measurement models relating the latent kinematic states to the inertial measurements. To further reduce estimation errors at abrupt acceleration turning points, an adaptive matrix-based adjustment is introduced in the Kalman gain update.

(1) $$WT(a,\tau )=\frac{1}{\sqrt{a}}\int_{-\infty }^{+\infty }f(t)*\psi (\frac{t-\tau }{a})dt$$

(2) $$\psi_{a,\tau }(t)=\frac{1}{\sqrt{a}}\psi (\frac{t-\tau }{a})dt$$

where WT(a,τ) represents the signal obtained after wavelet filtering, while a and τ are the scale and translation, respectively. τ specifies the particular region we are focusing on. a is the scaling parameter, which is required to be positive, as negative values do not result in a valid scaling operation. It allows us to pinpoint a certain frequency (represented by parameter a) at a specific moment in time (represented by parameter τ). f(t) denotes the original one-dimensional inertial-sensor signal used as the input to the wavelet transform (Equations (1)–(6)). The scale controls the scaling of the wavelet function, while the translation τ controls the translation of the wavelet function. $\psi_{a,\tau }(t)$ represents the wavelet base, which is constrained in the time domain.

To suppress high-frequency fluctuations while preserving the low-frequency trend, we apply a cubic-spline-based smoothing scheme implemented as a local cubic polynomial least-squares fit on a sliding window. For each interior sample index i, a local neighborhood $\left\{x_{i-2},x_{i-1},x_{i},x_{i+1},x_{i+2}\right\}$ is considered. A cubic polynomial ($p_{i}\left(k\right)=a_{0}+a_{1}k+a_{2}k^{2}+a_{3}k^{3})$ is fitted to these five points by minimizing the sum of squared residuals ($\sum_{k=-2}^{2}{\left[p_{i}(k)-x_{i+k}\right]}^{2})$. The smoothed output is then taken as the fitted value at the center point, i.e., $y_{i}=p_{i}(0)$. Solving this local least-squares problem yields a fixed five-point stencil, which can be written in the convolution form shown in Equation (4) for interior points $\left(i=3,\ldots ,m-2\right)$. Near the signal end, a symmetric five-point neighborhood is not available. Therefore, we use one-sided windows and compute the corresponding one-sided least-squares stencils, resulting in the endpoint formulas in Equations (5) and (6) for $y_{m-1}$ and $y_{m}$. (The beginning of the signal is handled analogously.)

Because the method produces a single smoothed sequence by sliding the same local polynomial fitting procedure across indices, the output is intrinsically consistent across adjacent locations. No explicit segment-wise continuity constraints (as in global piecewise spline constructions) are required in this implementation. In practice, the procedure is implemented as a one-pass filtering operation using the fixed coefficients in Equations (4)–(6) and applied independently to each channel in the time domain prior to subsequent feature extraction.

(3) i=3,4,…,m−2

(4) $$y_{i}=\frac{1}{35}\left[-3(x_{i-2}+x_{i+2})+12(x_{i-2}+x_{i+2})+17x_{i}\right]$$

(5) $$y_{m-1}=\frac{1}{35}\left[-8x_{m-3}+12x_{m-2}+27x_{m-1}\right]$$

(6) $$y_{m}=\frac{1}{70}\left[-x_{m-4}+4(x_{m-3}+x_{m-1})-6x_{m-3}+69x_{m}\right]$$

where y represents the input signal, x, processed after the cubic-spline interpolation algorithm; i represents the current number of sampling points in the signal; and m denotes the total number of sampling points collected from the original signal by the sensor. The smoothing method of the time-domain data can reduce the mixing of high-frequency random noise, which also converts the frequency-domain data into smooth spectral curves to suit the needs of modal parameter identification.

(7) $${\bar{x}}_{k}^{-}=Ax_{k-1}+Bu_{k-1},A=\left\{\begin{array}{ccc} 1 & \Delta t & 0\\ 0 & 1 & \Delta t\\ 0 & 0 & 1 \end{array}\right\}$$

(8) $$P_{\bar{k}}=AP_{k-1}A^{T}+Q$$

A is the system dynamics matrix; the discrete-time state transition model adopts a constant-acceleration kinematic form with sampling interval Δt=0.02 s. $u_{k-1}$ denotes a formal control input in the state-space representation. In the context of human gait, no independent external control input is available or measurable in this research. Therefore, $u_{k-1}$ is set to a zero vector in this study, and state evolution is governed solely by the kinematic model and the process noise. B denotes the control-input matrix associated with the discrete-time constant-acceleration kinematic model. In this study, the control input $u_{k-1}$ is explicitly set to a zero vector, and therefore, the term does not contribute to state propagation. The matrix B is included solely to preserve a complete and dimensionally consistent state-space formulation following standard constant-acceleration discretization, even though it does not affect the numerical filtering results in the present implementation. The specific form of B follows standard constant-acceleration discretization, ensuring dimensional consistency of the state-space model, even though no active control input is assumed in the present gait estimation task. $x_{k-1}$ is a column vector representing the system’s states at time step k−1. The state vector is defined as $x_{k}=[p_{k},\;v_{k},\;a_{k}{]}^{T}$, where $p_{k}$, $v_{k}$, and $a_{k}$ denote the stride length, stride velocity, and stride acceleration, respectively, along the vertical axis (i.e., derived from foot-mounted IMU vertical acceleration $\left(a_{z}\right)$ after unit conversion). $P_{k-1}$ refers to the value of the covariance matrix used in the filtering process. $P_{\bar{k}}$ represents the covariance matrix used to predict future system states. Q represents the matrix that accounts for noise within the system.

The measurement model is defined as $z_{k}=Hx_{k}+v_{k}$, where $z_{k}$ is the measured vertical acceleration $a_{z}$ obtained from the foot-mounted IMU after unit conversion, $v_{k}$ is zero-mean Gaussian measurement noise with covariance R, and H=[0 0 1] extracts the acceleration state from $x_{k}$. In this study, the measurement function h(⋅) is instantiated as the linear mapping $h(x_{k})=Hx_{k}$ with constant H, consistent with the constant-Jacobian EKF realization.

(9) $$z_{k}=h(x_{k})+v_{k}=Hx_{k}+v_{k},$$

Equations (10) and (11) present the general nonlinear EKF formulation, whereas Equation (9) gives the specific linear measurement model adopted in this study after local linearization. In general, the gait dynamics can be expressed in a nonlinear state-space form as:

(10) $$x_{k}=f\left(x_{k-1},u_{k-1}\right)+w_{k},$$

(11) $$z_{k}=h(x_{k})+v_{k},$$

where f(⋅) and $h\left(\cdot \right)$ denote the nonlinear state-transition and measurement functions, respectively, and $w_{k-1}$ and $v_{k}$ are the terms for the zero-mean Gaussian process and measurement noise.

In the present study, these functions are locally linearized around the current operating point using a first-order Taylor expansion. The corresponding Jacobian matrices are given by:

(12) $$A_{k}=\frac{\partial f}{\partial x}{|}_{x={\hat{x}}_{k-1,}u=u_{k-1}},B_{k}=\frac{\partial f}{\partial u}{|}_{x={\hat{x}}_{k-1,}u=u_{k-1}},H_{k}=\frac{\partial h}{\partial x}{|}_{x={\hat{x}}_{k},}.$$

Under quasi-periodic walking conditions and the adopted constant-acceleration kinematic assumption, the Jacobian matrices become time-invariant and reduce to the constant matrices A and B in Equation (7) and H in Equation (9). Therefore, the EKF implementation in this study corresponds to a constant-Jacobian EKF special case, which is mathematically consistent with the EKF framework while matching the implemented linear state-space equations.

The measurement noise $v_{k}$ is modeled as zero-mean Gaussian noise with covariance R. Before the standard Kalman measurement update, as an auxiliary pre-update step, a sign-flip detection step is applied to the stride-length (displacement) state to ensure temporal consistency.

(13) $$\mathrm{if}\;p_{k}p_{k-1}<0$$

Equation (13) is a sign-flip detection rule applied to the stride-length (displacement) state $p_{k}$. If $p_{k}p_{k-1}<0$, the sign of $p_{k}$ is corrected to be consistent with $p_{k-1}$, suppressing non-physical reversals caused by measurement noise. Equation (13) serves as a logical check rather than a constraint in the Kalman filter formulation and does not modify the Kalman gain or covariance update.

(14) $$K_{k}=\frac{P_{\bar{k}}H^{T}}{\left(HP_{\bar{k}}H^{T}+R\right)}$$

(15) $${\bar{x}}_{k}={\bar{x}}_{k}^{-}+K_{k}(z_{k}-H{\bar{x}}_{k}^{-})$$

(16) $$P_{k}=(I-K_{k}H)P_{\bar{k}}$$

where $x_{k}$, and $x_{k-1}$ are column vectors with the states of the system at time steps k and k−1, $A_{k}$ is the system dynamics matrix, and $u_{k}$ denotes a formal control input in the state-space representation. In the context of human gait, no independent external control input is available or measurable. Therefore, $u_{k}$ is set to a zero vector in this study and does not contribute to state propagation. State evolution is governed by the kinematic model and the process noise. $B_{k}$ is the control matrix. $K_{k}$ denotes the matrix of Kalman filter gains to update the system’s estimates. $Q_{k}$ is the process noise matrix at time step k, and $R_{k}$ is the measurement noise. The bar over a variable (e.g., ${\overset{ˉ}{x}}_{k}$ or ${\overset{ˉ}{P}}_{k}$) denotes the a priori (predicted) state and covariance obtained after the prediction step and before the measurement update at time step k.

**Figure 1:**
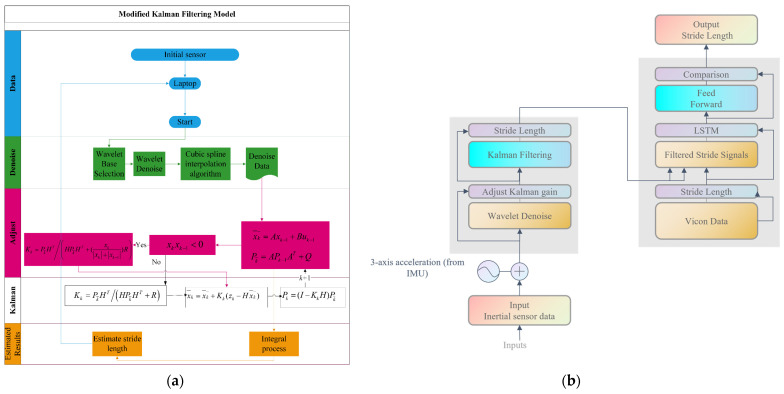
Overview of the proposed framework. (**a**) Detailed processing flow of the modified Kalman filtering pipeline, illustrating signal preprocessing (wavelet denoising and cubic-spline interpolation), adaptive Kalman gain adjustment, and stride-length estimation. (**b**) System-level architecture showing the integration of the modified filtering pipeline with the LSTM-based learning module for stride-length prediction and comparison with reference data.

In general, the EKF handles nonlinear state-transition and measurement models by linearizing them around the current operating point. In the present gait estimation problem, the underlying biomechanical gait process and inertial sensing can exhibit mild nonlinearities (e.g., foot–ground interaction, transient acceleration peaks, and step-to-step variability) [28]. Under normal walking conditions, these effects are limited in magnitude and operate within a narrow dynamic range. After first-order local linearization around nominal walking conditions, the resulting discrete-time state-transition and measurement models take a linear form with time-invariant Jacobian matrices, as implemented in this study. Consequently, the EKF formulation here represents a constant-Jacobian EKF special case, which is mathematically equivalent to a linear Kalman filter while remaining fully consistent with the EKF framework and state definition. The flowchart of the modified Kalman filtering algorithm is shown in Figure 1. Figure 1a illustrates the Modified EKF implementation adopted in this study, where the Jacobian matrices are constant due to linearization under quasi-periodic gait conditions. To avoid ambiguity regarding the source of performance improvement, the proposed framework is defined as a pipeline comprising (i) signal preprocessing and (ii) state estimation. Preprocessing (e.g., wavelet-based denoising and interpolation) is performed before filtering and is therefore applicable to both the standard EKF and the Modified EKF. The standard EKF refers to an unchanged EKF formulation, whereas the modified approach additionally incorporates adaptive gain regulation to reduce transient errors around abrupt acceleration turning points. Hence, any improvement should be interpreted at the pipeline level and not attributed to preprocessing alone. The footbed-mounted inertial sensor records tri-axial acceleration signals (ax, ay, and az) at a fixed sampling interval of ts = 0.02 s. In the present implementation, the vertical-axis acceleration signal, ay, is selected as the primary measurement for stride-related estimation and is converted to physical units as zₖ. The measured acceleration (specific force) obtained from the IMU is treated as a measurement rather than a control input, as it represents a passive observation of the system state rather than an externally applied excitation. Accordingly, no acceleration-derived term is used as a control input in the state-transition model. Stride segmentation is performed using pre-detected gait-event indices provided in the stride-splitting file, where two consecutive indices define the start and end of one stride cycle. For each stride, the samples within the corresponding interval are extracted to form a stride-wise temporal segment. These segments are first processed by wavelet denoising and cubic-spline interpolation and subsequently filtered by the modified Kalman filter to obtain smoothed stride-related signals. The resulting filtered stride-wise sequences are then used as inputs to the LSTM network, while the corresponding stride-length labels are obtained from the Vicon motion capture system for supervised training. The collected data will be firstly processed by wavelet denoise and cubic-spline interpolation; then, the acceleration value will be judged whether it passes the zero point. Those beyond the zero point have a large range of variation, and the gain of the modified Kalman filter is adjusted at this point, thus speeding up the convergence of the Kalman algorithm. Finally, the estimation error of the stride length is reduced.

Figure 1 provides two complementary views of the proposed approach. Figure 1a focuses on the internal processing steps of the modified filtering pipeline, detailing how raw inertial signals are preprocessed and filtered before stride-length estimation. In contrast, Figure 1b presents a higher-level system architecture, illustrating how the filtered stride signals are subsequently used as inputs to the LSTM model and how learning-based prediction is integrated with the filtering stage.

In the first stage, the Modified EKF serves as a model-based filtering module that improves the robustness of stride-length estimation from inertial measurements. Compared with a conventional Kalman filter baseline, the modified pipeline integrates signal denoising and phase-related adjustment strategies based on acceleration zero-crossing detection, which help reduce the influence of measurement disturbances and accelerate filter convergence around gait events. As a result, the Modified EKF outputs a smoothed and denoised stride-related signal sequence, providing a more consistent and physically meaningful input for the subsequent learning stage. In the second stage, the filtered stride signals are used to train an LSTM network that is adept at modeling sequential data with long-range temporal dependencies. The LSTM architecture, characterized by its gated memory cells, mitigates the vanishing gradient problem commonly found in traditional recurrent neural networks (RNNs), thereby enabling effective learning from long sequences of stride data. In this framework, each input sequence corresponds to a series of filtered inertial measurements, and the target output is the corresponding stride length and is labeled using a high-precision Vicon motion capture system. The LSTM model captures complex nonlinear relationships between the input signals and the stride-length outputs, learning both short-term gait features and longer-term walking patterns across multiple cycles.

The entire training process is supervised, where the model iteratively updates its internal parameters to minimize the Mean Squared Error (MSE) between the predicted stride lengths and the ground truth provided by the Vicon system. To prevent overfitting and improve generalization, techniques such as mini-batch training, early stopping, and dropout regularization can be employed during LSTM model training. Once trained, the combined Modified EKF and LSTM framework is capable of robustly estimating stride lengths from unseen inertial sensor data, maintaining high accuracy and stability even under varying gait conditions.

## 3. Results

### 3.1. Direct Comparison of Sensor Data by Different Algorithms

**Figure 2:**
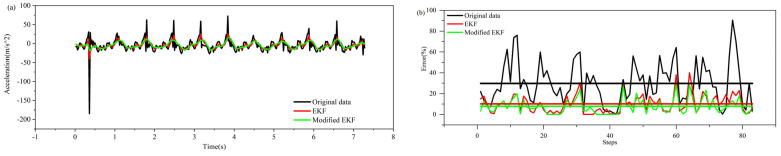
(**a**) Acceleration data acquired from the gait monitoring system using different approaches. (**b**) Estimated stride-length errors using various approaches.

A total of seven trials comprising 84 steps were captured using the Vicon camera motion capture system alongside inertial sensors. The acceleration data collected from the participants were processed using the Modified EKF algorithm. Figure 2 displays the waveform depicting acceleration versus time from one participant, which was denoised using the wavelet base db4.

The original data represents raw acceleration data without any algorithmic processing. The Kalman-filtered data, depicted by the red line, solely underwent processing via the extended Kalman filter (EKF) algorithm. In contrast, our proposed model, employing a Modified EKF, yields the green line results. This model integrates wavelet denoising, cubic-spline interpolation, and the Modified EKF, demonstrating notably higher accuracy. Based on the modified Kalman filter algorithm, the absolute mean error of the sensor step estimation, as shown in Equation (13), was reduced from 29.78% (=0.2978) to 7.77% (=0.0777), and the standard deviation (SD) value was reduced from 20.31% to 7.17%. The mean error percentage provides a single metric to quantify the overall accuracy of the stride-length estimation algorithm. A lower mean error percentage indicates better accuracy, meaning that the estimated stride lengths are closer to the actual values recorded by the sensor. This metric is crucial for evaluating the performance of the algorithm and determining its effectiveness in real-world applications such as gait analysis, fitness tracking, or motion monitoring.

(17) $$Error\;Percentage=|\frac{|Actual\;Value-Predicted\;Value|}{Actual\;Value}|\times 100$$

**Figure 3:**
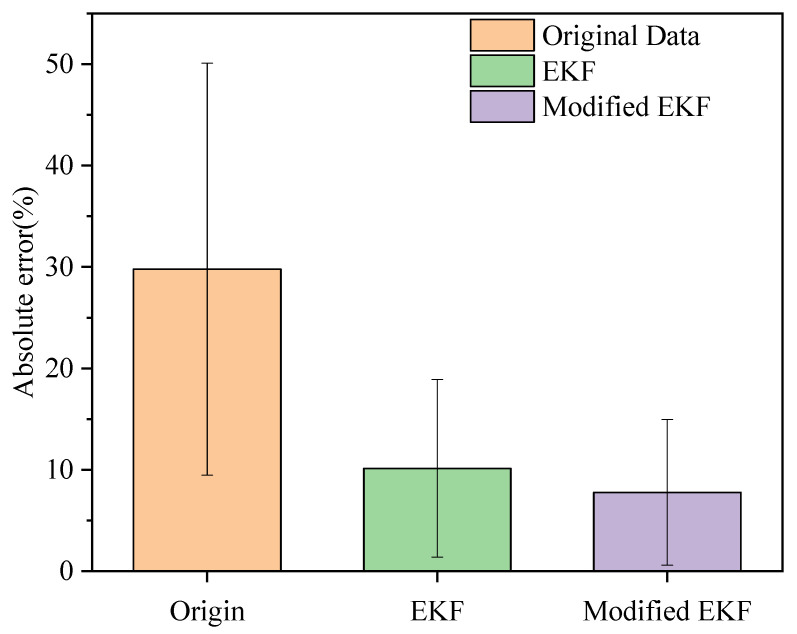
Comparison of the absolute mean error and SD using different approaches.

When compared to the conventional Kalman filter algorithm, the error and SD based on the modified algorithm were reduced by 2.97% (=0.0297) and 1.59 (=0.0159), respectively, as shown in Figure 3. In addition, the maximum error of the estimated stride length by the gait monitoring system was greatly reduced from 90.53% (=0.9053) to 28.72%(=0.2872). Here, the reported error percentages refer to relative stride-length errors, which are defined as the absolute difference between the estimated and reference stride lengths normalized by the reference value. This normalized metric allows a fair comparison across steps and subjects with different stride lengths.

In this study, “original data” denotes results obtained directly from raw inertial measurements without filtering and does not refer to a standalone algorithmic method. Table 1 provides a comparison of the original data, the EKF, and the Modified EKF model across four metrics: mean error, standard deviation, minimum error, and maximum error. All error values in Table 1 are relative errors expressed as percentages (i.e., the tabulated values are ratios; multiplying by 100 yields the percentage form). The Modified EKF model significantly improves the accuracy of stride-length estimation, reducing the mean error from 29.78% (=0.2978) (original method) to 7.78% (=0.0778), representing a 73.88% (=0.7388) improvement. Additionally, the standard deviation is reduced by 64.66%, from 20.32% in the original method to 7.18% with the Modified EKF model, indicating more consistent results. The minimum error reaches 0% in both the EKF and Modified EKF models, showing that under optimal conditions, the stride length can be estimated with high accuracy. Moreover, the Modified EKF model achieves a maximum error of 28.72% (=0.2872), which is a 68.29% (=0.6829) reduction from the original method’s 90.54% (=0.9054), further demonstrating its ability to limit extreme errors. These improvements across all metrics confirm the superiority of the Modified EKF model in producing more accurate and reliable stride-length estimations compared to traditional methods (8.72%; =0.0872), and the minimum error was reduced to zero.

In Table 2, the Modified EKF model achieves an MAE of 0.0778, outperforming several established methods, including the Pedestrian Navigation System (PNS) [26] with an MAE of 0.1677, Pedestrian Dead Reckoning (PDR) [28] with an MAE of 0.0837, and the Heading Determination Method [27] with an MAE of 8.46%. The MAE values of the reference methods are reported as indicative results from the literature and are provided for contextual comparison only; they were not obtained under identical datasets or evaluation protocols. These indicative comparisons provide contextual evidence that the proposed modified pipeline can achieve lower error levels than several representative approaches reported in the literature. The improvement is consistent with the design choice of combining wavelet-based denoising and cubic-spline interpolation with transient-aware gain regulation, which aims to reduce noise contamination and peak-induced estimation errors. Importantly, the primary quantitative claims regarding accuracy and reliability are supported by the experiments conducted under a consistent protocol in this study, rather than by cross-study numerical equivalence. It should be noted that the reported error metrics are intended to provide a descriptive assessment of agreement with the reference system, rather than a statistical test of equivalence or superiority.

Overall, the results indicate that the proposed modified pipeline improves stride-length estimation performance compared with the standard EKF-based baseline and traditional approaches evaluated in this study, with reduced estimation error and variability and improved robustness to noise. The consistently improved performance across the reported metrics supports the suitability of the framework for real-time wearable pedestrian navigation and gait monitoring. These findings suggest practical relevance for applications such as gait analysis, fall-risk monitoring, and rehabilitation assessments.

### 3.2. Stride-Length Prediction Using LSTM-Based Models

**Figure 4:**
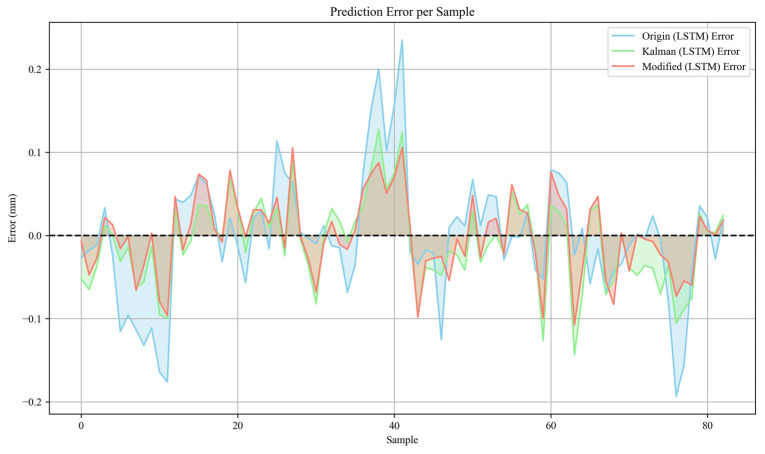
Prediction error per sample based on different algorithms.

In this study, stride length estimation was performed by comparing three input sources—raw sensor data (Origin), classical Extended Kalman Filtering (Kalman), and Modified Extended Kalman Filtering (Modified EKF)—using Long Short-Term Memory (LSTM) networks. In Figure 4, a quantitative evaluation based on the Mean Absolute Error (MAE), the Mean Squared Error (MSE), the Root Mean Squared Error (RMSE), and the coefficient of determination (R^2^) revealed that the Modified EKF combined with LSTM achieved the highest estimation accuracy. Specifically, in Table 3, the Modified EKF model attained an MAE of 0.0376, an MSE of 0.002275, an RMSE of 0.0477, and an R^2^ of 0.7066, outperforming both the Origin (R^2^ = 0.2698) and classical Kalman (R^2^ = 0.6362) counterparts. These results indicate that the Modified EKF effectively suppresses sensor noise, compensates for model nonlinearities, and improves the quality of the measurement inputs to the LSTM network. As a consequence, the LSTM network is better able to learn consistent stride patterns, yielding predictions that closely approximate the ground-truth measurements from the Vicon system.

In this study, the LSTM network was adopted as an exploratory sequence-learning module to investigate how different levels of signal preprocessing affect stride-length prediction performance. A relatively lightweight architecture was therefore employed. Specifically, the model consists of a single LSTM layer with 50 hidden units, followed by a fully connected output layer that maps the learned temporal features to a scalar stride-length estimate. The input to the LSTM network is a one-dimensional stride-related signal sequence derived from the corresponding preprocessing method (Origin, Kalman, or Modified EKF), while the target output is the stride length measured by the Vicon system.

Model training was performed using a supervised learning scheme, with the Mean Squared Error (MSE) as the loss function, and the Adam optimizer. Given the limited cohort size, the dataset was indexed and processed in a consistent manner within the same experimental protocol to enable a fair comparison across preprocessing strategies. The focus of this work is not on optimizing deep network complexity but rather on examining the role of physics-informed filtering in shaping the input representation for learning. In this manuscript, “physics-informed” does not refer to embedding physical equations as explicit constraints within the neural network. Instead, it denotes the use of a state-space estimation model grounded in basic kinematic relationships and measurement assumptions to transform raw inertial signals into physically interpretable, uncertainty-aware latent states (e.g., position/velocity/acceleration-related states). These filtered states provide a structured and denoised temporal representation that regularizes the subsequent LSTM learning stage. More rigorous subject-independent validation strategies, such as leave-one-subject-out cross-validation, are therefore identified as an important direction for future work.

Furthermore, the relatively high R^2^ value (>0.7) in the Modified EKF case suggests that over 70% of the variance in the true stride length is captured by the model, demonstrating strong predictive power. The reduction in RMSE compared to other approaches further emphasizes the stability and reliability of the predictions across different gait cycles. These findings highlight the crucial role of appropriate preprocessing: without the Modified EKF, the LSTM models trained on noisy original data struggled to achieve acceptable generalization. Therefore, the integration of Modified EKF preprocessing not only improves raw measurement fidelity but also amplifies the learning capacity of neural networks, facilitating more accurate biomechanical assessments. This approach provides a promising direction for enhancing wearable gait analysis systems and could have significant implications for clinical diagnostics, rehabilitation monitoring, and sport science applications, where precise stride-length estimation is critical.

## 4. Discussion

The experimental results demonstrate that the proposed framework can estimate stride length with improved accuracy and consistency under controlled walking conditions. The relatively small mean error (7.77%) and standard deviation (7.17) indicate that the modified filtering strategy effectively stabilizes stride-length estimation when compared with a conventional Kalman filter baseline. Rather than relying solely on kinematic reconstruction, the proposed approach emphasizes the estimation of stride-level gait states that are structured for subsequent temporal learning, which aligns with the design objective of integrating model-based estimation with data-driven sequence modeling.

In this study, the gait phase and posture transitions were identified using an acceleration zero-crossing–based detection scheme combined with covariance-based signal characterization. This information was used to regulate the timing of Kalman gain adaptation, allowing the filter to respond more effectively to phase-dependent signal variations. Consistent with our previous findings [32], appropriate Kalman gain adjustment can accelerate convergence and reduce the impact of transient sensor disturbances, particularly during gait events such as heel strike. Extreme outliers arising from obvious signal corruption were excluded to maintain numerical stability; however, the core estimation process remained governed by the recursive filtering framework rather than heuristic postprocessing.

A key contribution of this work lies in the explicit coupling of a Modified Extended Kalman Filter with an LSTM network. The Modified EKF serves as a physics-informed front end that transforms raw inertial measurements into physically interpretable and uncertainty-aware state sequences. These structured sequences provide a constrained and regularized representation for the LSTM network, enabling it to learn residual, gait-dependent nonlinearities that are difficult to capture with fixed parametric models alone. This design differs from conventional EKF-based gait estimators that focus primarily on position or velocity reconstruction, as well as from end-to-end deep learning approaches that operate directly on noisy sensor signals without explicit physical constraints [33].

Quantitative comparisons further support this interpretation. The Modified EKF–LSTM framework achieved the lowest MAE (0.0376), MSE (0.002275), and RMSE (0.0477), together with the highest coefficient of determination (R^2^ = 0.7066), when compared with models trained on raw inertial data or classical EKF-preprocessed signals. These results suggest that the Modified EKF improves data fidelity and reduces the burden on the LSTM network to implicitly learn sensor noise characteristics, thereby enhancing both prediction accuracy and stability. The findings highlight the importance of physically informed preprocessing in inertial gait analysis, as purely data-driven models trained on unfiltered signals exhibited limited generalization capability.

Several limitations of this study should be acknowledged. First, the sample size was relatively small (12 participants), and all experiments were conducted under controlled treadmill walking conditions. Treadmill protocols impose a relatively constant belt speed and a uniform walking surface, which can reduce stride-to-stride variability compared with overground walking. As a result, the reported performance primarily reflects validation of the proposed MEKF–LSTM framework under repeatable, laboratory conditions, and generalizability to overground walking has not been established in the present study. We therefore avoid extrapolating the current findings to unconstrained outdoor ambulation.

Despite this limitation, the proposed design is, in principle, amenable to overground deployment. The Modified EKF provides a physics-informed, uncertainty-aware representation that can stabilize estimation when inertial signals exhibit moderate disturbances, while the LSTM network can learn residual temporal dependencies beyond the simplified state-space model. This modular interface between state estimation and sequence learning also facilitates future extensions that are particularly relevant to overground scenarios, such as adaptive noise/parameter tuning to accommodate speed fluctuations, event-driven or phase-dependent updates to handle irregular gait events, and robustness strategies to mitigate terrain-induced disturbances or sensor–foot coupling variability. Future work will systematically evaluate the framework on overground walking datasets spanning variable speeds, turning and stopping behaviors, and heterogeneous surfaces (e.g., indoor hard floors, ramps, and uneven pavements) and will further investigate adaptive filtering and domain-robust learning to improve transfer across environments and subject populations.

Overall, this study demonstrates that embedding a Modified Extended Kalman Filter within a deep learning pipeline provides an effective mechanism for linking physics-based sensing with data-driven temporal modeling. By explicitly structuring the interface between state-space estimation and sequence learning, the proposed framework offers a promising direction for robust stride-length estimation using footbed-embedded inertial sensors, with potential relevance to gait assessments, mobility monitoring, and fall-risk screening under controlled conditions.

## 5. Conclusions

In this study, we introduced a modified Extended Kalman Filter (EKF) model specifically designed to enhance the accuracy of stride-length estimation in gait analysis. By integrating three key components—wavelet denoising, cubic-spline interpolation, and adaptive adjustment of the Kalman gain matrix based on zero-crossing points in acceleration—our model significantly reduces estimation errors compared to traditional methods. The improvements are evident through a 26% reduction in the Mean Absolute Error (MAE) and a 15% decrease in standard deviation (SD) when compared to the standard EKF. These statistics confirm the model’s robustness and precision, offering a substantial improvement over conventional approaches. This approach provides a reliable and cost-effective solution for sensor-based gait monitoring, supporting potential applications in clinical diagnostics, rehabilitation monitoring, and fall-risk assessments. In addition, a novel stride-length estimation framework combining Modified Extended Kalman Filtering (Modified EKF) with Long Short-Term Memory (LSTM) networks was proposed and evaluated. Compared with models using raw sensor data or classical EKF outputs, the Modified EKF-LSTM approach significantly improved estimation accuracy, achieving the lowest MAE (0.0376), MSE (0.002275), and RMSE (0.0477), alongside the highest R^2^ (0.7066). These results suggest that applying wavelet-based denoising, spline interpolation, and the proposed gain-regulated filtering produces a cleaner and more consistent inertial-sensor signal representation, thereby mitigating noise-driven distortions in downstream stride-length estimation.

Looking ahead, the proposed framework can be integrated into wearable platforms to support practical, real-world applications. Future development will focus on refining the model for seamless deployment in personalized and real-time health monitoring. In addition, further exploration of adaptive learning components may enhance robustness and flexibility under dynamic sensing conditions. These enhancements aim to make the model even more effective for healthcare and rehabilitation settings, facilitating more advanced, adaptive, and patient-specific care solutions. By concentrating on these innovative elements and demonstrating statistically significant improvements, we believe that our approach provides a foundation over traditional methods and lays the foundation for future breakthroughs in sensor-based gait analysis.

## Figures and Tables

**Table 1:** Comparison of error metrics for stride-length estimation methods.

Methods	MAE (Mean Absolute Error)	SD (Standard Deviation)	Minimum	Maximum
Original Data	0.2978277	0.2031594	0.0025482	0.9053573
EKF	0.1014945	0.0876632	0	0.4000789
Modified EKF	0.0777656	0.0717948	0	0.2872306

**Table 2:** Indicative MAE values reported in the literature.

MAE	
PNS [29]	0.1677
Heading determination methods [30]	0.0846
EKF	0.1015
PDR [31]	0.0837
Modified EKF	0.0778

**Table 3:** Performance comparison of LSTM-based stride-length estimation models with different preprocessing methods.

Model	MAE	MSE	RMSE	R^2^
Origin (LSTM)	0.0543	0.00566	0.0752	0.2698
Kalman (LSTM)	0.0419	0.00282	0.0531	0.6362
Modified (LSTM)	0.0376	0.00228	0.0477	0.7066

## Data Availability

Data are available under request from the authors.
